# Effects of different protein sources on fermentation metabolites and nutrient digestibility of brachycephalic dogs

**DOI:** 10.1017/jns.2017.46

**Published:** 2017-08-29

**Authors:** Maria Isabel Gonzalez Urrego, Laura Fantucci de O. Matheus, Karine de Melo Santos, Mariane Ceschin Ernandes, Mariana Monti, Danilo Ferreira de Souza, Júlio Cesar de Carvalho Balieiro, Lúcio Francelino Araújo, Cristiana F. Ferreira Pontieri, Márcio Antonio Brunetto

**Affiliations:** 1 Faculty of Animal Science and Food Engineering (FZEA), University of São Paulo (USP), Pirassununga, São Paulo, Brazil; 2 School of Veterinary Medicine and Animal Science (FMVZ), University of São Paulo (USP), Pirassununga, São Paulo, Brazil; 3 Grandfood Indústria e Comércio LTDA, Dourado, São Paulo, Brazil

**Keywords:** Brachycephalic dogs, Protein fermentation, Wheat gluten, Protein, CTTAD, coefficient of total tract apparent digestibility, HP, hydrolysed protein, PM, poultry meal, WG, wheat gluten

## Abstract

Benefits to microbial fermentation in the colon and as a consequence less flatulence can be promoted for the health of adult dogs according to the amount and protein source. The present study evaluated different protein sources in dry food for brachycephalic dogs regarding microbial fermentation and nutrient digestibility. Four dry dog foods with similar protein content were formulated for adult maintenance: poultry meal (PM) diet; wheat gluten (WG) diet; PM + WG diet; and PM + WG + hydrolysed protein (HP) diet. Eight French bulldog adult dogs were arranged in a 4 × 4 Latin square design during the 28 d trial. Fresh faeces were collected for assessment of nutrient digestibility and analyses of faecal pH, SCFA, biogenic amines, ammonia and lactate. Means were compared by the PROC MIXED procedure of SAS and by Tukey's test, considering *P* ≤ 0·05. The animals fed the WG and PM + WG diets showed higher digestibility for DM (*P* < 0·05), organic matter (*P* < 0·05), crude protein (*P* < 0·001) and lower faeces production (*P* < 0·02) than the PM and PM + WG + HP diets. Feeding diet PM + WG + HP resulted in lower faecal score and pH (*P* < 0·05) compared with other diets. Concentrations of fermentation metabolites were not statistically significantly different among diets. In conclusion, WG alone or in combination with PM improved protein and DM digestibility. Fermentation products were not affected by protein source.

Several factors may influence the amount of protein that reaches the colon after feeding, such as the amount of DM ingested and content and digestibility of protein sources included in the diet. Therefore, the use of highly digestible protein sources results in less flow of protein residues in the large intestine and a possible reduction in production of fermentation metabolites and flatulence in dogs^(^^1^^,^^2^^)^.

Although digestion and absorption of proteins in the small intestine are efficient processes, substantial amounts of undigested proteins are directed to the large intestine^(^^3^^,^^4^^)^, where the microbial fermentation of these components results in the production of various putrefaction compounds, such as ammonia, phenols, indoles, SCFA, branched-chain fatty acids, gases (H_2_, CO_2_ and methane), biogenic amines (putrescine, cadaverine, histamine, phenylethylamine) and lactate. Some of these compounds influence faecal odour and can be toxic if produced in high concentrations^(^^5^^,^^6^^)^. Moreover, according to Zentek *et al*.^(^^7^^)^, the ingestion of diets with high concentrations of proteins favours the growth of undesirable bacteria, such as *Clostridium perfringens*, and decreases the faecal counts of other beneficial bacteria, resulting in imbalance in the intestinal microbiota and consequent increase in excretion of enterotoxins and other metabolic products related to the increase of the protein decomposition in the colon.

Brachycephalic dog breeds such as French bulldogs have been identified as having faeces with a stronger odour and higher frequency of flatulence. Their typical anatomical characteristics result in aerophagia, predisposing to increased gas production. This gas production is associated with a higher presence of non-assimilated substrates, resulting in flatulence and foul-smelling faeces^(^^8^^,^^9^^)^. Degradation of undigested proteins in the colon may be responsible for the strong faecal odour in brachycephalic dogs. Therefore, nutritional strategies based on sources and altered concentrations of protein may be important in reducing the impact of fermentative activity in the colon and thus modulate the composition of the intestinal microbiota. Its metabolic activity and the formation of fermentation products are effects that can be important for the reduction of strong faecal odour in brachycephalic dogs. Zentek^(^^10^^)^ reported that dogs fed with higher digestible proteins had lower amounts of protein in the ileal chyme which would allow the decrease in putrefaction in the posterior intestine, and consequently reduction of the compounds involved in the gases and bad faecal odour. Thus, the improvement of protein quality and the use of different protein sources in dog food will decrease the fermentation products and as a consequence the odour of faeces in dogs of the French bulldog breed.

The objective of the present study was to evaluate the effects of inclusion of various protein sources in dry dog diets on the digestibility of nutrients and faecal fermentation products in adult brachycephalic dogs.

## Material and methods

The present study was conducted at the Nutrition Development Center of PremieR Pet, Dourado, São Paulo, Brazil, jointly with the Department of Animal Production and Nutrition at the School of Veterinary Medicine and Animal Science of the University of São Paulo, Pirassununga, São Paulo, Brazil. All care procedures were approved by the Ethics Research Committee on Animal Use of PremieR Pet (CEUA PremieR Pet – protocol no. 028-14).

### Animals, facilities and experimental design

Eight healthy adult French bulldog dogs (one male and seven female), intact and neutered, mean weight of 11·09 (sd 2·35) kg, mean age of 2·75 (sd 1·98) years and body condition score between 4 and 5 (9-point body condition score by Laflamme^(^^11^^)^) were used. Health status was confirmed before the beginning of the experiment by physical, blood and coproparasitological examinations. Dogs were previously dewormed and were up to date with vaccinations. The dogs were housed individually in kennels with solarium (11·2 m^2^). The animals were distributed in replicate 4 × 4 Latin squares, four treatments (diets) and four periods, totalling eight replicates per treatment. The treatments were balanced by the animals’ body weight. Each experimental period lasted 28 d. The animals were adapted to the diet for 20 d; followed by 5 d of faecal collection for apparent digestibility and faecal score; and 3 d of fresh faeces collection to determine the fermentation products.

### Diets

Four extruded isonutrient diets were produced to meet the adequate intake of the requirements for the maintenance of adult dogs^(^^12^^)^, containing: brewer's rice, beet pulp, cellulose, chicken fat, fish oil, egg powder, brewer's yeast, palatability enhancer, potassium chloride, mineral–vitamin premix, magnesium oxide, salt, dicalcium phosphate, calcium carbonate and antioxidant. The diets presented contained 13 % of protein from different sources (poultry meal (PM); wheat gluten (WG); PM + WG (50 % PM and 50 % WG); PM + WG + liver hydrolysed protein (HP) (PM, WG and HP, with 33·33 % inclusion of each)). The diets’ proximate analyses composition means were approximately: 23 % protein, 16 % fat, 3 % crude fibre, 6 % ash, 7 % moisture, 54 % N-free extract and 3·9 kcal/kg (16·3 kJ/kg). To verify if the extrusion processing conditions were able to cook the starch, the degree of gelatinisation of the starch was determined by the amyloglucosidase method described by Sá *et al*.^(^^13^^)^

### Food intake and digestibility experiment

The total faecal collection method was used to perform the coefficients of total tract apparent digestibility (CTTAD) assay^(^^14^^)^, consisting of an initial phase of 20 d of adaptation to the diet, followed by 5 d of faecal collection. The animals were fed twice per d (07·00 and 16·30 hours) and received water *ad libitum*. The amount of food offered and refused was recorded at each meal. The amount of food offered was calculated by formula: 130 × body weight^0·75^ = kcal/d (544 × body weight^0·75^ = kJ/d), based on the energy requirement prediction equation for maintenance of active adult dogs^(^^12^^)^. The food offered was weekly adjusted to keep the animals’ body weight stable.

The faeces were individually collected, weighed and kept in a freezer (−20°C) for further analysis. After the collection period, the faecal samples were thawed, homogenised, and pooled for each animal and period. Faecal samples were dried in a forced-air oven (320SE; Fanem) at 55°C for 72 h. Dried faeces and feed samples were then ground in a cutting mill with a 1 mm screen sieve (MOD 340; ART LAB). The qualitative analysis of the faeces was determined over the stool collection period for digestibility, scoring from 1 (watery stools) to 5 (very hard and resected stools)^(^^15^^)^. Based on laboratory results, the CTTAD of DM, crude protein, ether extract in acid hydrolysis, organic matter, N-free extract and gross energy were calculated according to the equation of Pond *et al*.^(^^16^^)^

Diets and faeces were submitted to DM, crude protein, ether extract in acid hydrolysis, crude fibre and ash analyses according to the Association of Official Analytical Chemists (AOAC)^(^^17^^)^. Gross energy was determined in a bomb calorimeter (1281; Parr Instrument Company). All analyses were conducted in duplicate and were repeated when CV was greater than 5 %.

### Fermentation metabolites

Fresh faeces were collected up to 30 min after defecation. Faecal pH was determined by digital pH meter (DM-20; Digimed) in a solution of faeces and distilled water (2 g/18 ml)^(^^18^^)^. Lactic acid concentration was measured by spectrophotometry at 565 nm (QUICK-Lab; DRAKE Eletrônica Comércio LTDA)^(^^19^^)^. Faeces were diluted in distilled water (3 g/9 ml).

For SCFA and branched-chain fatty acid determinations, 3 g faeces were diluted in 9 ml of 16 % formic acid, kept in a refrigerator, homogenised daily and centrifuged for 15 min at 15°C and 5000 rpm. This procedure was repeated three times using only the supernatant fraction and stored in a freezer (−15°C). SCFA and branched-chain fatty acids were determined by GC, according to methodology described in the literature^(^^20^^)^.

The biogenic amine profile was determined using 0·5 g faeces preserved in 7 ml of 5 % trichloroacetic acid. The samples were then centrifuged and filtered according to Vale & Gloria^(^^21^^)^. Identification of the amines was performed by HPLC (Shimadzu Corporation).

Ammonia concentration was determined using 3 g faeces acidified with 9 ml of 16 % formic acid. The samples were centrifuged and stored according to the methodology described for SCFA determination. Aliquots of 2 ml were diluted in 13 ml distilled water and distilled in N distillation. The distillation was carried out with 5 ml of 2 m-potassium hydroxide solution and the titration with hydrochloric acid (0·005 mol/l)^(^^22^^)^.

### Statistical analysis

The data were analysed considering a duplicate 4 × 4 Latin square design. Treatments were compared by ANOVA and, in case of significant effects, we used Tukey's test for *post hoc* group comparisons. The analyses were performed by PROC MIXED, using version 9.3 of SAS software^(^^23^^)^. The model contemplated the treatment as fixed effect, and the animal and the period as random effects. Statistical significance was set at *P* < 0·05.

## Results

The body weight of the animals did not change during the study. Food intake did not differ between diets (*P* > 0·05; Table 1). The PM, WG, PM + WG and PM + WG + HP diets presented, respectively, 91·0, 87·4, 98·5 and 77·7 % gelatinisation of starch. Animals fed the PM + WG diet had a greater CTTAD for DM (*P* < 0·05) and those fed the WG diet for organic matter (*P* < 0·05). A higher CTTAD for crude protein was observed for the WG and PM + WG diets compared with the PM and PM + WG + HP diets. Feeding the PM + WG + HP diet resulted in lower faecal score and pH compared with other diets. Also faecal volume was smaller with the PM + WG diet compared with the other diets (g faeces/100 g food on a DM basis). However, faecal fermentation metabolites did not differ among diets (Table 2).

**Table 1.** Nutrient intake, coefficients of apparent total tract digestibility, metabolisable energy and faecal traits of French bulldogs fed experimental diets with different protein sources (Mean values and pooled standard errors; *n* 8 dogs per diet)

	Diet						
Item	PM	WG	PM + WG	PM + WG + HP	sem	CV (%)	*P*
Body weight (kg)	11·4	11·5	11·3	11·5			
Nutrient intake (g/kg body weight per d)							
Natural matter	16·93	17·84	18·10	19·39	0·76	22·91	0·2234
DM	15·86	16·47	16·93	17·97	0·71	22·85	0·3127
Organic matter	14·98	15·63	16·16	17·12	0·68	22·91	0·2652
Ash	0·88	0·83	0·77	0·85	0·04	22·86	0·2756
Crude protein	3·48	4·17	3·95	3·96	0·17	23·54	0·0923
Fat	2·63	2·65	2·93	3·10	0·12	23·46	0·0627
N-free extract	8·63	8·29	8·90	9·56	0·38	22·95	0·2133
Crude energy (kcal/kg body weight per d)*	72·86	76·37	77·07	82·95	3·28	22·85	0·2923
Apparent total tract digestibility (%)							
DM	85·22^b,c^	87·35^a,b^	88·77^a^	86·16^b^	0·37	2·31	0·0234
Organic matter	88·08^b,c^	89·61^a^	90·44^a,b^	88·28^b^	0·31	1·89	0·0237
Ash	36·84	44·64	42·99	43·08	1·51	19·42	0·2791
Crude protein	83·98^b^	88·08^a^	88·53^a^	83·62^b^	0·54	3·37	0·0001
Fat	96·75	96·47	96·92	96·56	0·13	0·71	0·1600
N-free extract	91·71	92·09	93·28	91·78	0·27	1·61	0·1706
Crude energy	88·31	89·58	90·19	89·70	0·47	2·84	0·5671
Faecal traits							
Faecal pH	6·37^a^	6·39^a^	6·44^a^	6·14^b^	0·05	4·21	0·0190
Faecal score	4·34^a^	4·46^a^	4·38^a^	3·98^b^	0·07	9·06	0·0064
Faeces							
g faeces/d (as fed)	77·68	69·63	67·48	85·88	3·16	22·55	0·2158
g faeces/d (DM basis)	82·96	74·38	74·24	91·49	0·94	20·94	0·0664
g faeces/100 g food (as fed)	42·34	35·60	35·44	40·04	1·18	16·60	0·1103
g faeces/100 g food (DM basis)	13·84^a,b^	11·68^b,c^	10·97^c^	12·80^b^	0·35	15·22	0·0206

PM, poultry meal; WG, wheat gluten; HP, hydrolysed protein.

^a,b,c^ Mean values within a row with unlike superscript letters were significantly different (*P* < 0·05; Tukey's test).

* To convert kcal to kJ, multiply by 4·184.

**Table 2.** Fermentation products: lactic acid, faecal ammonia, SCFA, branched-chain fatty acids (BCFA) and biogenic amines of French bulldogs fed experimental diets with different protein sources (Mean values and pooled standard errors; *n* 8 dogs per diet)

	Diet						
Item	PM	WG	PM + WG	PM + WG + HP	sem	CV (%)	*P*
Lactic acid (mmol/kg faecal DM)	11·38	11·65	16·59	12·82	1·14	47·06	0·2372
Faecal ammonia (mmol/kg faecal DM)	207·05	167·80	174·65	194·24	6·95	20·19	0·1078
SCFA (mmol/kg faecal DM)							
Acetic	92·47	97·73	101·9	96·97	4·05	22·43	0·9088
Propionic	65·53	65·37	60·51	73·15	2·98	24·27	0·2502
Butyric	3·56	3·17	2·91	3·31	0·21	34·52	0·2952
Total SCFA	141·37	167·82	165·33	151·75	6·56	21·21	0·8318
BCFA (mmol/kg faecal DM)							
Valeric	0·68	0·62	0	0·75	–	–	–
Iso-valeric	4·93	4·46	4·36	4·45	0·28	32·77	0·5805
Iso-butyric	21·43	20·73	21·88	20·65	1·09	27·84	0·9896
Total BCFA	23·15	26·78	22·96	22·24	1·22	25·50	0·9715
Biogenic amines (mg/100 g faecal natural matter)							
Tiramine	1·14	1·43	1·64	1·19	0·13	53·19	0·6454
Putrescine	6·28	4·71	6·79	8·50	0·40	32·22	0·0960
Cadaverine	0·75	0·90	0·77	1·00	0·10	61·46	0·7458
Spermidine	2·67	2·28	2·95	2·60	0·14	28·24	0·2182
Feniletilamine	0·23	0·00	0·48	1·35	–	–	–
Triptamine	0·23	0·22	0·20	0·21	0·02	53·40	0·9235
Total amines	11·30	9·54	12·83	14·85	0·79	32·79	0·9758

PM, poultry meal; WG, wheat gluten; HP, hydrolysed protein.

## Discussion

The crude protein CTTAD of the four experimental diets were high (Table 1), indicating good utilisation of the protein sources evaluated in the study. However, higher CTTAD were observed in the diets formulated with higher proportions of WG (WG and PM + WG diets). Stool production was greatest in the PM treatment, having a possible relationship with the low CTTAD of the protein, besides the other nutrients. Nery *et al*.^(^^2^^)^ evaluated diets formulated with PM and WG, together (WPMP) and separated and in different concentrations (WGLP: inclusion of 22 % dietary protein; WGHP: inclusion of 39 % dietary protein; WPMP: 29 % dietary protein). These authors found higher coefficients of apparent digestibility of protein in WGLP (86·6 %), in WPMP (91·4 %) and WPMP (86·2 %) comparing with only PM (82·2 %) diets. In the present study, the WG-based diet was formulated with 21 % protein content and 13 % of the total protein on a DM basis came from gluten. The results of protein digestibility for the WG (88·08 %) and PM + WG (88·5 %) diets were similar or even better compared with the study cited. Partially, these best results may be related to the quality of the ingredients used in food formulation. However, the lower apparent digestibility coefficients of the PM + WG + HP diet, which were not expected, could be explained by lower starch gelatinisation (77·7 %). The influence of starch gelatinisation on improving protein digestibility has been studied by Loureiro *et al*.^(^^24^^)^

Some authors^(^^25^^,^^26^^)^ have reported that the ideal faecal score and pH values are expected with higher-quality protein at lower concentrations. This results in a reduction of colonic fermentation similar to that observed in the present study. Faecal scores did not differ among PM, WG and PM + WG treatments, but were similar to previous research with several breeds and sizes of dogs^(^^6^^)^. This previous study found a high water content in the faeces of dogs fed with PM and lower moisture in the faeces of dogs fed diets containing WG. The low-protein fermentation in the present study is probably related to the lower flow of undigested proteins in the large intestine, due to the low protein content in the diets and the high digestibility of the same ones.

The study presented some limitations related to the lack of information in the literature about digestibility in brachycephalic dogs, and the use of WG in pet food as a protein source. These limitations restricted the discussion and references in the study. Also, the inclusion of a non-brachycephalic breed as a control in the study might have demonstrated differences between the breeds in this paper.

### Conclusion

The WG used as the sole source of protein or in combination with PM increased protein digestibility. However, this effect was not able to alter the fermentation parameters measured.
